# Effects of GLP-1 Receptor Agonists on Bone Mineral Density in Patients with Type 2 Diabetes Mellitus: A 52-Week Clinical Study

**DOI:** 10.1155/2021/3361309

**Published:** 2021-09-17

**Authors:** Ting-ting Cai, Hui-qin Li, Lan-lan Jiang, Hui-ying Wang, Meng-hui Luo, Xiao-fei Su, Jian-hua Ma

**Affiliations:** Department of Endocrinology, Nanjing First Hospital, Nanjing Medical University, Jiangsu, China

## Abstract

**Introduction:**

Hypoglycemic drugs affect the bone quality and the risk of fractures in patients with type 2 diabetes mellitus (T2DM). We aimed to investigate the effects of glucagon-like peptide-1 receptor agonists (GLP-1RAs) and insulin on bone mineral density (BMD) in T2DM.

**Methods:**

In this single-blinded study, a total of 65 patients with T2DM were randomly assigned into four groups for 52 weeks: the exenatide group (*n* = 19), dulaglutide group (*n* = 19), insulin glargine group (*n* = 10), and placebo (*n* = 17). General clinical data were collected, and BMD was measured by dual-energy X-ray absorptiometry.

**Results:**

Compared with baseline, the glycosylated hemoglobin (HbA1c) decreased significantly in the exenatide (8.11 ± 0.24% vs. 7.40 ± 0.16%, *P* = 0.007), dulaglutide (8.77 ± 0.37% vs. 7.06 ± 0.28%, *P* < 0.001), and insulin glargine (8.57 ± 0.24% vs. 7.23 ± 0.25%, *P* < 0.001) groups after treatment. In the exenatide group, the BMD of the total hip increased. In the dulaglutide group, only the BMD of the femoral neck decreased (*P* = 0.027), but the magnitude of decrease was less than that in the placebo group; the BMD of L1-L4, femoral neck, and total hip decreased significantly (*P* < 0.05) in the placebo group, while in the insulin glargine group, the BMD of L2, L4, and L1-4 increased (*P* < 0.05). Compared with the placebo group, the BMD of the femoral neck and total hip in the exenatide group and the insulin glargine group were increased significantly (*P* < 0.05); compared with the exenatide group, the BMD of L4 in the insulin glargine group was also increased (*P* = 0.001).

**Conclusions:**

Compared with the placebo, GLP-1RAs demonstrated an increase of BMD at multiple sites of the body after treatment, which may not exacerbate the consequences of bone fragility. Therefore, GLP-1RAs might be considered for patients with T2DM. This trial is registered with ClinicalTrials.gov NCT01648582.

## 1. Introduction

Type 2 diabetes mellitus (T2DM) is a global public health problem that is known to be associated with severe morbidity and mortality [1]. T2DM has been shown to have a negative impact on bone quality along with increasing the risk of fracture [2]. However, the exact cause of bone fragility in patients with T2DM is not clear and may be multifactorial due to chronic hyperglycemia, insulin resistance, obesity, inflammatory factors, and certain antidiabetic medications [1, 3, 4]. The potential role of the drugs used for the treatment of T2DM in abnormal bone metabolism has been attracting increasing attention [5].

Glucagon-like peptide 1 (GLP-1) is an intestinal peptide produced by intestinal epithelial L cells, which is mainly secreted after glucose intake or meal. GLP-1 combines with GLP-1 receptor, which can promote the synthesis and secretion of insulin and inhibit appetite. GLP-1 receptor agonists (GLP-1RAs) are widely used in T2DM as glucose-dependent glucose-lowering drugs [6]. Since GLP receptor is expressed in osteoblasts, GLP-1RAs may affect BMD. Zhang et al. found that GLP-1RAs might be beneficial to bone metabolism by stimulating the proliferation and differentiation of osteoblasts and reducing the accumulation of advanced glycation end products (AGEs) through the determination of bone markers, mRNA, and protein expression of receptors for advanced glycation end product (RAGE) in femur and morphological index of femur in mice fed with high-fat diet [7]; the reports on the impact of GLP-1RAs on fracture risk appears contradictory. Pereira et al. demonstrated that GLP-1RAs were associated with bone protection in ovariectomized mice [8]; however, some meta-analyses have shown that GLP-1RAs are not associated with a decreased bone fracture risk [9, 10]. Our findings may provide evidence for the effects of GLP-1RAs on BMD in patients with T2DM.

Currently, assessment of bone mineral density (BMD) using dual-energy X-ray absorptiometry (DEXA) is the standard diagnostic technique for the evaluation of bone strength and fracture risk [11, 12]. At present, there are few reports about the effect of GLP-1RAs on BMD. Therefore, we conducted a 52-week clinical trial to evaluate the effect of GLP-1RAs on BMD in patients with T2DM and to observe whether there were new fractures during the treatment.

## 2. Materials and Methods

### 2.1. Subjects

The single-blinded study was approved by the ethics committee of Nanjing First Hospital of Nanjing Medical University. All procedures were performed in accordance with the 1964 Helsinki Declaration, revised in 2013. Informed consent was obtained from all patients participating in the study. The study was conducted at Nanjing First Hospital of Nanjing Medical University from December 2012 to December 2016. All of the patients with T2DM were diagnosed by the admitting physician, and the diagnostic criterion of T2DM was according to the World Health Organization in 1999 [13].

Inclusion criteria were as follows: (1) patients with T2DM of at least 6 months duration; (2) age ≥ 40 years; (3) 6.5% ≤ HbA1c ≤ 10%; (4) the glycemic control was stable for at least 90 days (unadjusted hypoglycemic treatment).

The exclusion criteria were as follows: (1) type 1 diabetes mellitus; (2) gestational diabetes; (3) patients who had been treated with GLP1-Ras earlier; (4) hormone replacement therapy or administration of vitamin D and calcium agents that affect bone metabolism; (5) patients with long history of smoking and daily intake of >60 g of alcohol; (6) patients with any of the following diseases: severe acute and/or chronic complications of T2DM, including ketoacidosis or hyperosmolality, severe cardiovascular disease, end-stage renal disease, severe infectious disease, severe diabetic gastroparesis, or long-term use of drugs that directly affect the gastrointestinal motility, metabolic disease including hyperthyroidism, hypothyroidism, hypercortisolism, and hypopituitarism; (7) cancer metastasis to bone; and (8) patients with a history of glucocorticoid treatment.

A total of 70 patients with T2DM were included in this study and were assigned in a ratio of 2 : 2 : 1 : 2 to the following four treatment groups: exenatide (2 mg/week), dulaglutide (1.5 mg/week), insulin glargine (6 unit/day), and placebo (once a week). The two kinds of GLP-1RAs, exenatide and dulaglutide, have been widely used in China's diabetic population, and two drugs are all weekly preparations, which are more conducive to rigorous research. All research subjects maintained their original therapeutic drug type and dose throughout the research except adding one research drug by group. The whole study lasted for 52 weeks.

### 2.2. Data Collection

The clinical data of the patients were collected. Data regarding glycosylated hemoglobin (HbA1c), total cholesterol (TC), triglycerides (TG), blood urea nitrogen (BUN), and serum creatinine (Scr) were measured and recorded by trained staff before and after 52 weeks of treatment. HbA1c was the secondary endpoint of this study.

The BMD values of the lumbar vertebrae (L), femoral neck, and total hip were measured using DEXA (GE lunar prodigy, Ge, Madison, Ma, USA; coefficient of variation = 0.30%) before and 52 weeks after treatment in all patients. The endpoint of this study was to assess the changes of BMD, especially the total hip of BMD.

### 2.3. Statistical Methods

Statistical analyses were conducted using the SPSS 20.0 statistical package. The continuous variables were tested for normal distribution, and the variables with normal distribution were expressed as means ± standard error mean (SEM). Nonnormally distributed data were presented as interquartile ranges (IQR). One-way ANOVA, a nonparametric test, and chi-square test were used to analyze the differences between the four groups at baseline and within the group before and after treatment. Age was used as a nuisance covariate to analyze the differences between the four groups. The least significant difference (LSD) was used for multiple comparisons. Statistical analysis was bilateral, and *P* < 0.05 was considered significant.

## 3. Results

### 3.1. Baseline Characteristics

A total of 70 patients were recruited. However, five patients dropped out of the trial and did not complete the trial. Finally, 19 patients (nine men and 10 women) in the exenatide group, 19 patients (11 men, eight women) in the dulaglutide group, 10 patients (seven men, three women) in the insulin glargine group, and 17 patients (nine men, eight women) in the placebo group were included. The adherence of patients who completed the whole study in each group was 100%. As shown in Table 1, there were no significant patient differences with regard to sex, age, weight, body mass index (BMI), HbA1c, BMD, and other biochemical indicators among the four groups.

### 3.2. Clinical Data and BMD Measurement before and after 52 Weeks within the Groups

During the whole study, no fracture occurred in the patients. Compared with baseline, the HbA1c of exenatide group (8.11 ± 0.24% vs. 7.40 ± 0.16%, *P* = 0.007), dulaglutide (8.77 ± 0.37% vs. 7.06 ± 0.28%, *P* < 0.001), and insulin glargine group (8.57 ± 0.24% vs. 7.23 ± 0.25%, *P* < 0.001) decreased significantly after treatment. In the placebo group, the HbA1c increased after treatment compared with baseline, but the difference was not statistically significant (8.01 ± 0.23% vs. 8.15 ± 0.30%, *P* = 0.632).

After 52 weeks of treatment, the BMD of L1-L4, femoral neck, and total hip showed a decrease in the placebo group (*P* < 0.05). However, in the exenatide group, the BMD of the total hip increased (0.95 ± 0.03 g/cm^2^ vs. 1.03 ± 0.04 g/cm^2^, *P* = 0.02), while in the insulin glargine group, the BMD of L2 and L4 increased (*P* < 0.05) (Table 2) (Supplementary Figure 1).

### 3.3. Intergroup Comparisons of the Changes in Each Index after Treatment

Table 3 shows the differences in the parameters before and after 52 weeks of treatment between the four groups. Compared with patients in the placebo group of HbA1c levels, those in the other three groups decreased significantly (*P* < 0.05). Compared with placebo group, the BMD of L1 (0.02 ± 0.01 g/cm^2^ vs. −0.04 ± 0.02 g/cm^2^, *P* = 0.014), L1-4 (0.05 ± 0.07 g/cm^2^ vs. −0.12 ± 0.06 g/cm^2^, *P* = 0.017), femoral neck (0.09 ± 0.05 g/cm^2^ vs. −0.11 ± 0.04 g/cm^2^, *P* = 0.005), and total hip (0.09 ± 0.03 g/cm^2^ vs. −0.12 ± 0.04 g/cm^2^, *P* = 0.001) in the exenatide group was relatively increased. Similarly, compared with the placebo group, the BMD of L2, L4, femoral neck, and total hip in the insulin glargine group was also increased significantly (*P* < 0.05); it is noteworthy that the BMI of the exenatide group was relatively lower than insulin glargine group (Supplementary Figure 2).

## 4. Discussion

Our study results revealed a significant decrease in the BMD of the placebo group after 52 weeks of treatment; however, in the exenatide group, the BMD of the total hip increased. In the dulaglutide group, only the BMD of the femoral neck decreased, but the magnitude of the decrease was less than that in the placebo group. In comparison, the BMD of the L2, L4, and L1-4 vertebrae demonstrated an increase in the insulin glargine group. This study demonstrated that the two common GLP-1RAs, exenatide and dulaglutide, have a positive effect on BMD and may not exacerbate the consequences of bone fragility.

Currently, it is known that a variety of factors may be related to the increased risk of fractures in T2DM, such as the duration of diabetes, blood glucose control, and hypoglycemic drugs [3, 14]. The use of hypoglycemic drugs in patients with T2DM is essential. Poor glycemic control can accelerate the occurrence of complications. At present, there are several types of hypoglycemic drugs, such as thiazolidinediones (TZDs), biguanides, GLP-1RAs, dipeptidyl peptidase-4 (DPP-4) inhibitors, and insulin. Previous studies indicated that different hypoglycemic drugs may have different effects on BMD. Montagnani et al. showed that TZDs could lead to bone loss [15, 16]. A clinical study by Borges et al. revealed that metformin had a neutral effect on bone density [17]; similarly, a meta-analysis by Gilbert et al. suggested that the effect of sulfonylureas on bone metabolism and BMD seemed to be neutral [18]. A meta-analysis conducted by Monami et al. found that DPP-4 inhibitors have a bone protective effect and prevent fractures [19].

It has been recognized that a higher BMD has a protective effect on obese individuals with impaired glucose metabolism or in patients who have recently been diagnosed with T2DM [20]. However, doctors advocate weight loss in most type 2 diabetes patients to reduce the cardiovascular risk, which may reduce the BMD and increase bone turnover.

In the present study, the exenatide and dulaglutide groups maintained the original bone density despite the reduction in the bodyweight when compared to the insulin glargine group, indicating that their maintenance effect of BMD was independent of the body weight. In the insulin glargine group, the BMD improved in multiple regions after 52 weeks of treatment compared to the pretreatment values (*P* < 0.05). This is consistent with the results of the previous studies and may be due to the reduction in bone resorption by insulin glargine [21]. Although there was no statistical difference in the weight, it showed an upward trend (from 70.30 ± 5.33 to 72.00 ± 5.18, *P* = 0.113). Therefore, GLP-1RAs may be a better choice to control blood glucose levels and lose bodyweight without reducing the BMD.

Some recent animal studies observed that GLP-1RAs could increase bone mass and BMD and thus have beneficial effects on bone health [14]. Mabilleau et al. found that compared with wild-type mice, GLP-1 receptor knockout mice had lower bone strength and higher bone resorption [22]. Eminov et al. revealed that exenatide treatment in ovariectomized rats reversed the significant decrease in BMD, trabecular number, trabecular thickness, and trabecular area, while showing a significant protective effect on trabecular bone microstructure [23].

Furthermore, some clinical studies have shown that GLP-1RAs have neutral or protective effects on bone health. Iepsen et al. observed that GLP-1RAs (liraglutide) could reduce the loss of bone mineral content and increase the levels of bone formation markers (procollagen type 1 N-terminal peptide and osteocalcin) in obese women [24]. A previous meta-analysis by Mabilleau et al. found that liraglutide and exenatide had a neutral effect on fracture incidence compared with other antidiabetic drugs [9]. In a 2-year prospective clinical study, Gilbert et al. demonstrated that liraglutide alone does not have a negative effect on total BMD in patients with type 2 diabetes [18].

In the current study, we assessed the changes in BMD between the groups and found that the BMD of the whole hip and other sites in the dulaglutide and exenatide groups showed an upward trend (*P* < 0.05) compared with the placebo group, which is consistent with the results of the above studies.

Different GLP-1RAs might have different effects on fractures when compared with placebo or other antidiabetic drugs. A meta-analysis by Cheng et al. reported that liraglutide significantly reduced the risk of fractures (odds ratio [OR] = 0.38, 95% confidence interval [CI] 0.17-0.87), whereas exenatide increased the risk of fractures (OR = 2.09, 95% CI 1.03-4.21) [25]. In this study, the effects of exenatide and dulaglutide on BMD were also different. While the BMD of the femoral neck decreased after 52 weeks of treatment in the dulaglutide group, this phenomenon was not observed in the exenatide group. This indicated that exenatide has better effect on maintaining bone mineral density than that of dulaglutide, which suggests that the effects of different GLP-1RAs on bone metabolism are indeed different; however, the specific mechanisms still need further study.

The effect of GLP-1RAs may promote osteogenic differentiation of bone marrow stromal cells by regulating *β*-catenin signal transduction [26] and increase the expression of osteoprotegerin (OPG) genes to affect the OPG/nuclear factor-*κ*B ligand-receptor activator (RANKL)/nuclear factor-*κ*B receptor activator (RANK) system to reverse the decrease in bone mass and increase bone formation [27, 28]. GLP-1 RAs can also reduce the number of osteoclasts and levels of serum bone resorption markers along with inhibiting bone resorption [26, 29]. In addition, parafollicular cells of the thyroid also express GLP-1 receptors, which can improve bone metabolism by reducing the parathyroid hormone, as continuous parathyroid hormone stimulation can promote bone resorption [7]. However, the specific molecular mechanism of GLP-1RAs has not been fully elucidated.

Unfortunately, the sample size of this study was only 70 subjects. Moreover, we did not detect markers related to bone metabolism in this study. The specific mechanism of the effect of hypoglycemic drugs on bone metabolism is still unknown. In the future, we need to expand the sample size and extend the follow-up time for further in-depth exploration.

In conclusion, GLP-1RAs have an effect of maintaining BMD and provide a treatment option for patients with osteoporosis and type 2 diabetes.

## Figures and Tables

**Table 1:** Characteristics of patients.

	Exenatide	Dulaglutide	Insulin glargine	Placebo	*P*
Cases (*n*)	19	19	10	17	
Male/female (*n*)	9/10	11/8	7/3	9/8	0.694
Age (years)	62.95 ± 1.70	57.42 ± 1.81	64.36 ± 2.93	62.00 ± 1.21	0.060
Body weight (kg)	70.37 ± 2.64	68.92 ± 2.42	70.30 ± 5.33	73.12 ± 2.84	0.780
BMI (kg/m^2^)	27.11 ± 0.91	25.34 ± 0.74	25.79 ± 0.94	27.04 ± 0.83	0.337
HbA1c (%)	8.11 ± 0.24	8.77 ± 0.37	8.57 ± 0.24	8.01 ± 0.23	0.265
TC (mmol/L)	4.18 ± 0.20	4.95 ± 0.23	4.69 ± 0.32	4.48 ± 0.19	0.082
TG (mmol/L)	2.11 ± 0.32	1.76 ± 0.19	1.68 ± 0.23	2.74 ± .0.47	0.471
BUN (mmol/L)	5.78 ± 0.53	5.62 ± 0.32	5.86 ± 0.23	5.59 ± 0.25	0.963
Scr (*μ*mol/L)	73.58 ± 4.21	72.58 ± 3.51	71.7 ± 6.98	69.18 ± 4.18	0.898
BMD-L1 (g/cm^2^)	0.98 ± 0.05	0.98 ± 0.03	1.02 ± 0.06	1.07 ± 0.04	0.287
BMD-L2 (g/cm^2^)	1.08 ± 0.05	1.09 ± 0.04	1.13 ± 0.07	1.14 ± 0.04	0.747
BMD-L3 (g/cm^2^)	1.15 ± 0.05	1.16 ± 0.04	1.20 ± 0.09	1.22 ± 0.04	0.673
BMD-L4 (g/cm^2^)	1.16 ± 0.06	1.18 ± 0.04	1.20 ± 0.08	1.22 ± 0.05	0.830
BMD-L1-L4 (g/cm^2^)	1.10 ± 0.05	1.11 ± 0.03	1.14 ± 0.07	1.17 ± 0.04	0.689
BMD-femoral neck (g/cm^2^)	0.85 ± 0.02	0.94 ± 0.03	0.93 ± 0.05	0.96 ± 0.03	0.090
BMD-total hip (g/cm^2^)	0.95 ± 0.03	1.01 ± 0.04	1.02 ± 0.06	1.07 ± 0.04	0.131

Abbreviation: BMI: body mass index; HbA1c: glycosylated hemoglobin; TC: total cholesterol; TG: triglycerides; BUN: blood urea nitrogen; Scr: serum creatinine; BMD-L: bone mineral density of lumbar vertebrae; BMD-femoral neck: bone mineral density of femoral neck; BMD-total hip: bone mineral density of total hip; data shown as mean ± SEM.

**Table 2:** Comparison of clinical data and BMD before and 52 weeks within groups.

	Exenatide			Dulaglutide			Insulin glargine			Placebo		
	Baseline	52 weeks	*P*	Baseline	52 weeks	*P*	Baseline	52 weeks	*P*	Baseline	52 weeks	*P*
Body weight (kg)	70.37 ± 2.64	68.79 ± 2.59	0.012^∗^	68.92 ± 2.42	69.00 ± 2.42	0.913	70.30 ± 5.33	72.00 ± 5.18	0.113	73.12 ± 2.84	72.09 ± 2.65	0.077
BMI (kg/m^2^)	27.11 ± 0.91	26.50 ± 0.88	0.012^∗^	25.34 ± 0.74	25.36 ± 0.74	0.929	25.79 ± 0.94	26.46 ± 0.96	0.088	27.04 ± 0.83	26.68 ± 0.80	0.083^∗^
HbA1c (%)	8.11 ± 0.24	7.40 ± 0.16	0.007^∗^	8.77 ± 0.37	7.06 ± 0.28	<0.001^∗∗^	8.57 ± 0.24	7.23 ± 0.25	<0.001^∗∗^	8.01 ± 0.23	8.15 ± 0.30	0.632
TC (mmol/L)	4.18 ± 0.20	4.11 ± 0.22	0.741	4.95 ± 0.23	4.73 ± 0.24	0.203	4.69 ± 0.32	4.59 ± 0.39	0.828	4.48 ± 0.19	4.86 ± 0.24	0.028^∗^
TG (mmol/L)	2.11 ± 0.32	2.29 ± 0.28	0.288	1.76 ± 0.19	1.87 ± 0.27	0.660	1.68 ± 0.23	2.17 ± 0.87	0.460	2.74 ± .0.47	2.42 ± 0.50	0.485
BMD-L1 (g/cm^2^)	0.98 ± 0.05	0.99 ± 0.05	0.238	0.98 ± 0.03	0.98 ± 0.03	0.788	1.02 ± 0.06	1.03 ± 0.05	0.525	1.07 ± 0.04	1.04 ± 0.04	0.053
BMD-L2 (g/cm^2^)	1.08 ± 0.05	1.07 ± 0.05	0.532	1.09 ± 0.04	1.09 ± 0.04	0.599	1.13 ± 0.07	1.16 ± 0.08	0.048^∗^	1.14 ± 0.04	1.12 ± 0.05	0.065
BMD-L3 (g/cm^2^)	1.15 ± 0.05	1.16 ± 0.06	0.480	1.16 ± 1.220.04	1.16 ± 0.04	0.869	1.20 ± 0.09	1.24 ± 0.09	0.051	1.22 ± 0.04	1.21 ± 0.05	0.399
BMD-L4 (g/cm^2^)	1.16 ± 0.06	1.17 ± 0.05	0.477	1.18 ± 0.04	1.16 ± 0.04	0.118	1.20 ± 0.08	1.24 ± 0.08	0.010^∗^	1.22 ± 0.05	1.22 ± 0.05	0.993
BMD-L1-L4 (g/cm^2^)	1.10 ± 0.05	1.15 ± 0.07	0.498	1.11 ± 0.03	1.11 ± 0.03	0.384	1.14 ± 0.07	1.17 ± 0.07	0.001^∗∗^	1.17 ± 0.0.9804	1.05 ± 0.04	0.045^∗^
BMD-femoral neck (g/cm^2^)	0.85 ± 0.02	0.93 ± 0.05	0.109	0.94 ± 0.03	0.93 ± 0.03	0.027^∗^	0.93 ± 0.05	0.93 ± 0.05	0.354	0.96 ± 0.03	0.85 ± 0.02	0.017^∗^
BMD-total hip (g/cm^2^)	0.95 ± 0.03	1.03 ± 0.04	0.020^∗^	1.10 ± 0.04	1.00 ± 0.04	0.104	1.02 ± 0.06	1.02 ± 0.06	0.628	1.07 ± 0.04	0.95 ± 0.03	0.014^∗^

Abbreviation: BMI: body mass index; HbA1c: glycosylated hemoglobin; TC: total cholesterol; TG: triglycerides; BMD-L: bone mineral density of lumbar vertebrae; BMD-femoral neck: bone mineral density of femoral neck; BMD-total hip: bone mineral density of total hip; data shown as mean ± SEM. Significant correlation values are shown in asterisk (^∗^*P* < 0.05, ^∗∗^*P* ≤ 0.001).

**Table 3:** Comparison of the difference to each index between groups after treatment for 52 weeks.

	Exenatide	Dulaglutide	Insulin glargine	Placebo		Exenatide vs. dulaglutide	Exenatide vs. insulin glargine	Exenatide vs. placebo	Dulaglutide vs. insulin glargine	Dulaglutide vs. placebo	Insulin glargine vs. placebo
	▲				*P*	*P*	*P*	*P*	*P*	*P*	*P*
Body weight (kg)	−1.58 ± 0.57	0.08 ± 0.71	1.70 ± 0.97	−1.03 ± 0.54	0.016^∗^	0.116	0.002	0.565	0.097	0.318	0.012^∗^
BMI (kg/m^2^)	−0.61 ± 0.22	0.02 ± 0.26	0.67 ± 0.35	−0.36 ± 0.20	0.010^∗^	0.110	0.001^∗^	0.479	0.068	0.371	<0.001^∗∗^
HbA1c	−0.71 ± 0.23	−1.70 ± 0.31	−1.34 ± 0.25	0.14 ± 0.28	<0.001^∗∗^	0.018^∗^	0.138	0.029^∗^	0.583	<0.001^∗∗^	0.002^∗^
TC (mmol/L)	−0.07 ± 0.20	0.71 ± 0.16	0.09 ± 0.41	0.38 ± 0.16	0.207	0.595	0.952	0.126	0.709	0.047^∗^	0.185
TG (mmol/L)	0.18 ± 0.17	1.12 ± 0.26	0.50 ± 0.84	−0.32 ± 0.44	0.611	0.910	0.622	0.347	0.210	0.422	0.210
BMD-L1 (g/cm^2^)	0.02 ± 0.01	0 ± 0.01	0.01 ± 0.02	−0.04 ± 0.02	0.077	0.478	0.770	0.014^∗^	0.765	0.084	0.076
BMD-L2 (g/cm^2^)	−0.01 ± 0.01	−0.01 ± 0.01	0.02 ± 0.01	−0.03 ± 0.01	0.043^∗^	0.898	0.070	0.183	0.107	0.157	0.005^∗^
BMD-L3 (g/cm^2^)	0.01 ± 0.02	0 ± 0.01	0.03 ± 0.01	−0.01 ± 0.02	0.332	0.862	0.476	0.203	0.417	0.285	0.083
BMD-L4 (g/cm^2^)	0.01 (-0.03, 0.03)	0.12 (0.03, 0.27)	0.24 (0.11, 0.40)	0.01 (-0.04, 0.03)	0.015^∗^	<0.001^∗∗^	0.001^∗∗^	0.653	0.343	<0.001^∗∗^	0.001^∗∗^
BMD-L1-L4 (g/cm^2^)	0.05 ± 0.07	−0.01 ± 0.01	0.03 ± 0.01	−0.12 ± 0.06	0.091	0.345	0.846	0.017^∗^	0.560	0.152	0.070
BMD-femoral neck (g/cm^2^)	0.09 ± 0.05	−0.01 ± 0.01	0.01 ± 0.01	−0.11 ± 0.04	0.003^∗^	0.062	0.206	0.005^∗^	0.741	0.056	0.049^∗^
BMD-total hip (g/cm^2^)	0.09 ± 0.03	−0.01 ± 0.00	0 ± 0.00	−0.12 ± 0.04	<0.001^∗∗^	0.049^∗^	0.077	0.001^∗∗^	0.944	0.006^∗^	0.021^∗^

Abbreviation: BMI: body mass index; HbA1c: glycosylated hemoglobin; TC: total cholesterol; TG: triglycerides; BMD-L: bone mineral density of lumbar vertebrae; BMD-femoral neck: bone mineral density of femoral neck; BMD-total hip: bone mineral density of total hip; data shown as mean ± SEM or median (first quartile, third quartile). Significant correlation values are shown in asterisk (^∗^*P* < 0.05, ^∗∗^*P* ≤ 0.001).

## Data Availability

The data used to support the findings of this study are available from the corresponding author upon request.

## Supplementary Materials

Supplementary Materials Supplementary Figure 1: (a)–(g) BMD (L1, L2, L3, L4, L1-4, femoral neck, and total hip) at baseline and after treatment within groups. Data are mean ± SEM. Supplementary Figure 2: (a)–(g) changes of BMD (L1, L2, L3, L4, L1-4, femoral neck, and total hip) between groups after treatment for 52 weeks. Data are mean ± SEM.
