# Supplementary material for: Effects of GLP-1 Receptor Agonists on Bone Mineral Density in Patients with Type 2 Diabetes Mellitus: A 52-Week Clinical Study
Source: Biomed Res Int. 2021 Sep 17;2021:3361309. doi: 10.1155/2021/3361309 (PMC8464416; doi:10.1155/2021/3361309)
Supplement: Supplementary Materials — Supplementary Figure 1: (a)–(g) BMD (L1, L2, L3, L4, L1-4, femoral neck, and total hip) at baseline and after treatment within groups. Data are mean ± SEM. Supplementary Figure 2: (a)–(g) changes of BMD (L1, L2, L3, L4, L1-4, femoral neck, and total hip) between groups after treatment for 52 weeks. Data are mean ± SEM. [file 3361309.f1.docx]

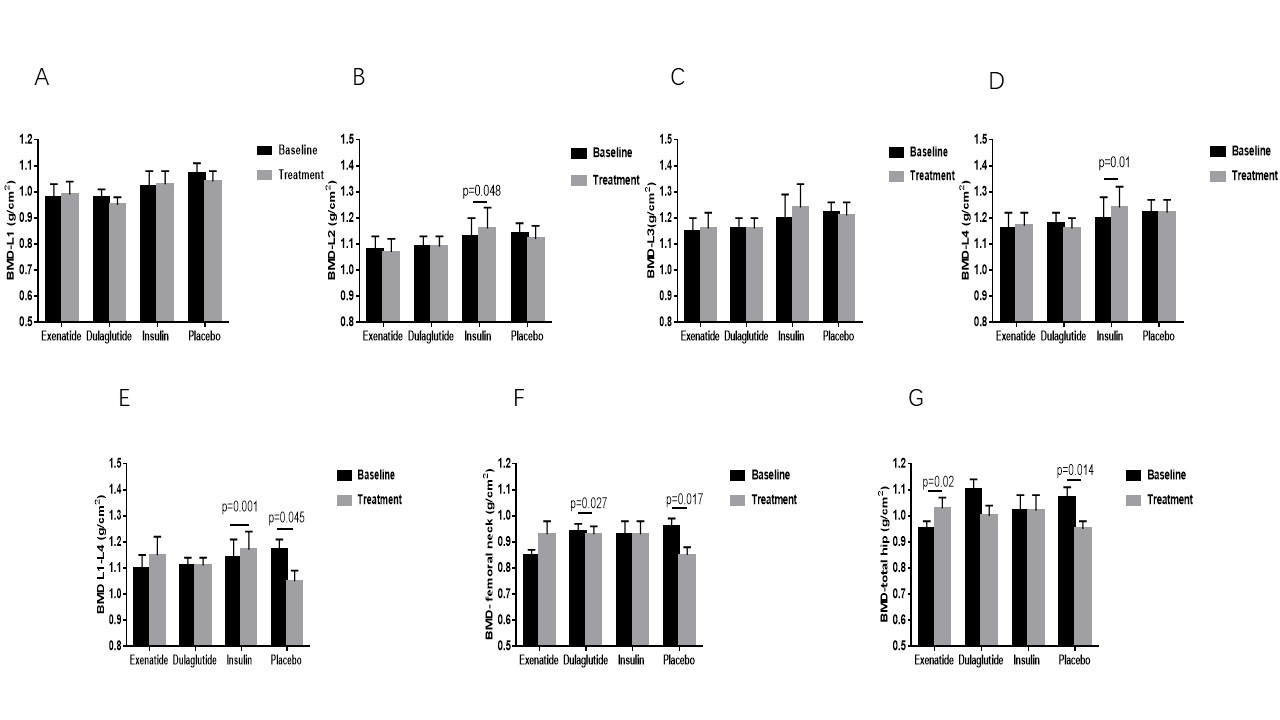


Supplementary Figure 1 A-G BMD (L1, L2, L3, L4, L1-4, femoral neck and total hip) at baseline and after treatment within groups. Data are mean ± SEM.


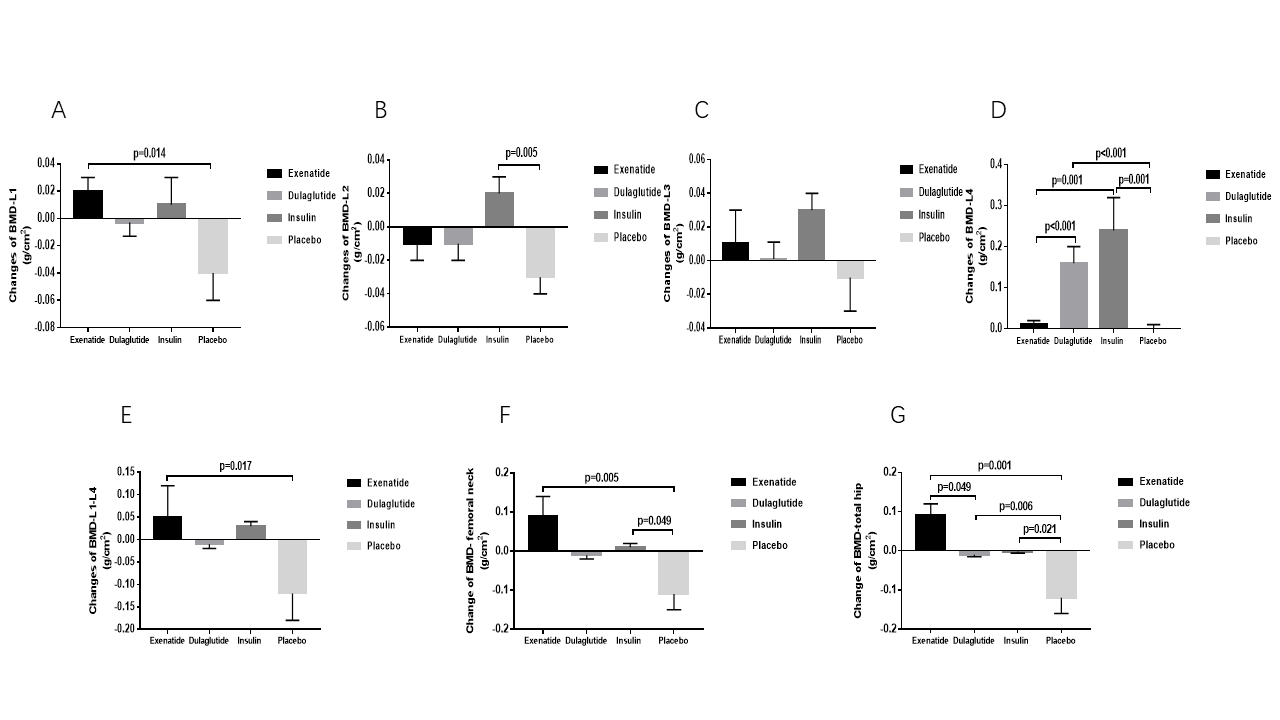


Supplementary Figure 2 A-G changes of BMD (L1, L2, L3, L4, L1-4, femoral neck and total hip) between groups after treatment for 52 weeks. Data are mean ± SEM.

.
